# Rheumatism and Gout

**Published:** 1903-01-31

**Authors:** 


### Rheumatism and Gout.

**Acute Rheumatism.**—T. McCrae [1] gives an analysis of 270 cases in the Johns Hopkins Hospital for 13 years. Fifty-five per cent. occurred in the months February to May, and in 75 per cent. the first attack came on before the thirtieth year of life. The average duration of the fever was twelve days, and in 10 patients out of 270 the attack began with tonsillitis. Thirty-eight per cent. showed no heart affection, while 32 showed definite organic lesions, and 13 per cent. had murmurs which disappeared under treatment. Albumen was found on one or many occasions in 50 per cent., death occurred in only three cases, and the average duration of an attack was 38 days. Walsh holds that salicylates should be given in daily amounts of 90 to 120 grains when they are well borne, but for heart complications alkalies are needed. Antipyrin may be used when the salicylates produce dyspnœa or other bad results, but 60 to 90 grains daily for three or four days will be required. E. M. Merrins [2] suggests that chronic joint changes are due to some long past microbic invasion, such as acute rheumatism, gonorrhœa, or typhoid, and that a chill is able to start local troubles in joints which have been thus enfeebled. So, too, in syphilis and leprosy joint affections occur many years after the original infections. Invading organisms are devoured by phagocytes, but some remain permanently as parasites in fixed cells, until after some chill they are able to overpower their hosts. Parkes Weber [3] would include the infantile articular form with enlargement of the glands and spleen under acute rheumatism because of the occurrence of endocarditis and pericarditis. It has been regarded by Sansom and others as a form of rheumatoid arthritis on account of the symmetrical swellings about the hands and feet. This swelling is due to an infiltration of the tissues, and not to effusion. Rolleston thinks it a septic state occurring in rachitic subjects. Payne and Poynton have failed to find their microbe in a case of this type, and it is clear that if the affection is truly rheumatic the disease must be remarkably modified by the age of the patient.

As a symptom of rheumatism in children, *epistaxis* is worth noting, according to Sidney Phillips.[4] It sometimes comes on with an acute attack, sometimes it alternates with articular affections. The absence of pain in the articular rheumatism of children is very common, and may cause the disease to be overlooked; but if epistaxis appears, the joints should be carefully examined for swellings and the heart for murmurs. Epistaxis has been given as one of the results of treatment by salicylates, but such cases are possibly due to the disease itself. This connection of epistaxis with rheumatism is not surprising when we recall the frequency of purpura in rheumatism.

*Myocarditis* of severe type is rare in rheumatism,[5] except when pericarditis is present. Theodore Fisher gives a fatal instance where extensive degeneration had taken place with slight evidence of pericarditis. He believes that the rheumatic poison can produce this degeneration, though in a less degree than the diphtheritic poison does. The disease of the heart muscle may go on advancing for a long period, since we know that the rheumatic organisms may live in the diseased valves for years. He describes also a case of advanced degeneration of the muscle and fibroid change probably due to rheumatism where the only symptoms were those due to chronic valve disease.

*Hyperpyrexia.*—Allen and J. W. Russell [6] record a case where the temperature reached 115° and the patient recovered after packing in ice. However, symptoms like those of disseminated sclerosis developed during his recovery and became permanent, though a slight improvement took place after a while. One or two instances have been recorded of similar nerve changes following rheumatic hyperpyrexia, but whether they are due to slight hæmorrhages or to inflammatory processes is unknown.

**Bacteriology.**—F. Meyer [7] obtained a streptococcus from cases of tonsillitis which had been followed by acute rheumatism, and injected this into rabbits. Symptoms of articular rheumatism were produced and 21 per cent. of the animals developed endocarditis. The organism was found in their joints and in the growths on the valves under the form of a diplococcus which took a lighter blue with the Gram method than ordinary streptococci do. They could not be obtained from the blood. If other organisms were injected along with them, they alone were found in the joints, but even here they were very short-lived and quickly disappeared. Poynton and Paine[8] hold that in "rheumatic" arthritis the primary seat of the rheumatic organism is in the synovial tissues, that the endothelium bars its entrance into the joint, and that when it does succeed in passing this, active phagocytosis takes place. Hence it is quickly destroyed, and therefore cultures from the joints are so often negative. In chronic cases a perivascular fibrosis occurred around the small blood-vessels, which reduced their lumen. In the infantile type of arthritis with enlarged glands there is much periarticular thickening, but it is doubtful what this disease really is. As to rheumatoid arthritis, many different infections may produce the symptoms; whether there is a special disease of this type is undetermined; so, too, whether the nervous affections and the joint lesions are the common result of toxins, or the joint lesions due to the nerve changes is unsettled. Rheumatoid arthritis may sometimes follow repeated attacks of acute rheumatism, and this may be due to an intensified local virulence of the poison and not to a second injection. Similarly, simple and malignant endocarditis are both at times the result of one poison. Herrick[9] reviews the subject of *pneumococcal arthritis* and finds that, including three cases of his own, there are 52 recorded instances. The affection is often monoarticular, and frequently some previous injury has weakened the joint chosen. It may come on during convalescence from pneumonia, or it may be primary, and on the other hand arthritis during an attack of pneumonia may be due to other causes. The joint itself or merely the periarticular structures, such as bursæ, may be the seat of the trouble. The constitutional symptoms vary remarkably; there may be very few due to the joint trouble, or profound septicæmia may come on. The mortality of the recorded cases was 65 per cent., due largely to the other organs attacked at the same time; as to treatment when pus is present he recommends an early incision, though spontaneous recovery, or one after aspiration, has occasionally occurred even in purulent cases. To explain the facts that the affection is most prone to occur during convalescence, Leroux refers to the law that attenuated microbes are apt to lodge on serous surfaces and in joints. The joints may either show only a serous synovitis, or thick pus with rapid and severe destruction of all the structures around.

[1] Med. Rec., June 21. [2] Med. Rec., March 22. [3] Lancet, April 19. [4] Lancet, Feb. 22. [5] Lancet. [6] Lancet, July 19. [7] Med. Rec., Nov. 1. [8] Lancet, Aug. 23. [9] Amer. Jour. Med. Sci., July.

(*To be continued.*)

